# Longitudinal trajectories of insulin resistance in women with polycystic ovary syndrome: a functional data analysis approach

**DOI:** 10.1530/EC-25-0266

**Published:** 2025-08-29

**Authors:** Fahimeh Ramezani Tehrani, Mohammad Fayaz, Faegheh Firouzi, Fereidoun Azizi, Maryam Tohidi, Samira Behboudi-Gandevani

**Affiliations:** ^1^ Reproductive Endocrinology Research Center, Research Institute for Endocrine Molecular Biology, Research Institute for Endocrine Sciences, Shahid Beheshti University of Medical Sciences, Tehran, Iran; ^2^ Foundation for Research & Education Excellence, Vestavia Hills, Alabama, USA; ^3^ Health and Social Medicine Department, School of Medicine, Shahed University, Tehran, Iran; ^4^ Endocrine Research Center, Research Institute for Endocrine Disorders, Research Institute for Endocrine Sciences, Shahid Beheshti University of Medical Sciences, Tehran, Iran; ^5^ Prevention of Metabolic Disorders Research Center, Research Institute for Metabolic and Obesity Disorders, Research Institute for Endocrine Sciences, Shahid Beheshti University of Medical Sciences, Tehran, Iran; ^6^ Faculty of Nursing and Health Sciences, Nord University, Bodø, Norway

**Keywords:** fasting insulin, functional data analysis approach, HOMA-IR, insulin resistance, polycystic ovary syndrome, trajectory

## Abstract

**Introduction:**

Insulin resistance (IR) plays a central role in the pathophysiology of polycystic ovary syndrome (PCOS), yet its long-term trajectory across different phenotypes and obesity statuses remains unclear. This study aimed to model longitudinal IR patterns in women with PCOS, those meeting an isolated PCOS criterion, and healthy controls.

**Methods:**

This population-based prospective study included 1,759 reproductive-aged women (18–49 years) from the Tehran Lipid and Glucose Study, categorized into PCOS (*n* = 287), isolated PCOS criterion (*n* = 536), and healthy control (*n* = 936) groups, stratified by BMI (<25 kg/m^2^, 25–29.99 kg/m^2^, and ≥30 kg/m^2^) and WC (<88 cm and ≥88 cm). Functional principal component analysis modeled HOMA-IR and fasting insulin level trajectories as sparse functional data.

**Results:**

Women with PCOS were younger (23.5 vs 25 and 28 years, *P* < 0.05) and had lower BMI than healthy controls (23.38 vs 24.46 kg/m^2^, *P* < 0.05), with no baseline differences in HOMA-IR. Both HOMA-IR and fasting insulin trajectories varied by obesity status. Women with PCOS and either obesity or central obesity showed a pronounced upward trend in IR over time, while those without general or central obesity exhibited more stable or improving patterns. In contrast, healthy and isolated criterion groups showed relatively stable or mildly increasing HOMA-IR and fasting insulin levels, with steeper increases in the presence of obesity.

**Conclusion:**

This study provides new insights into the diverse trajectories of IR among women with PCOS, those with an isolated PCOS criterion, and healthy controls. Progressive worsening of IR was observed in women with PCOS and obesity, while those with PCOS without obesity showed improvement. Healthy and isolated criterion groups exhibited more stable patterns.

## Introduction

Polycystic ovary syndrome (PCOS) is one of the most common endocrine disorders, affecting 5–20% of women of reproductive age worldwide (1, 2). It manifests through a range of clinical features, primarily including hyperandrogenism and/or hyperandrogenemia, oligo/anovulation (OA), and polycystic ovarian morphology (PCOM) (3). The etiology of PCOS is multifactorial, involving a complex interplay of genetic, epigenetic, and environmental factors, which contribute to its heterogeneous presentation (3, 4).

It is well documented that insulin resistance (IR) and obesity are strongly associated with PCOS (5, 6, 7). This association is particularly pronounced in women with hyperandrogenic PCOS phenotypes compared to those with the ovulatory or normoandrogenic phenotypes (8, 9, 10). IR exacerbates hyperandrogenism by acting as a gonadotropin in the ovary, stimulating adrenal androgen secretion, and altering luteinizing hormone pulsatility. In turn, hyperandrogenism further worsens IR, creating a vicious metabolic cycle (11). This cycle is further complicated by increased adiposity, particularly visceral fat, which contributes to systemic inflammation and metabolic stress. These factors further impair insulin signaling and exacerbate hormonal imbalances, which may lead to metabolic comorbidities, including type 2 diabetes and cardiovascular diseases (11, 12).

Nevertheless, IR and obesity are not universally present in PCOS, nor is PCOS consistently observed in women with IR or obesity (6, 8, 13, 14). The close association between PCOS and obesity is demonstrated by epidemiological data, indicating that 38–88% of women with PCOS are either overweight or obese (15, 16). Notably, those with elevated body mass index (BMI) often exhibit more severe reproductive symptoms and are therefore more likely to receive a PCOS diagnosis (16). In addition to potentially increased risk for obesity in PCOS, women with PCOS are also more likely to carry excess adiposity in the central body region compared with BMI-matched controls (17). Central obesity exacerbates IR, and this visceral fat deposition intensifies both metabolic and reproductive dysfunction in PCOS, contributing to hyperandrogenism, glucose intolerance, dyslipidemia, and hypertension (18, 19, 20). However, the patterns of IR across time, particularly among different PCOS phenotypes and obesity statuses, remain poorly understood and have yet to be thoroughly investigated.

Traditional methods for analyzing longitudinal data, such as local regression and generalized additive mixed models, have some challenges due to the dependent nature of repeated measurements and the frequent occurrence of sparse data in clinical settings, where subjects often have varying numbers of observations (21). Functional data analysis (FDA) offers a promising solution by treating the entire trajectory of a subject as a single functional entity, without assuming independence between time points (22). Within FDA, functional principal component analysis (FPCA) is a powerful tool for dimensionality reduction, particularly in sparse datasets, enabling the identification of underlying patterns and variations in longitudinal data (16). This approach is well-suited to capture the non-linear, phenotype-specific trajectories of IR in PCOS, which may be influenced by factors such as obesity and central fat distribution.

Therefore, the aim of this study was to investigate the longitudinal trajectory of IR, assessed by the homeostatic model assessment of IR (HOMA-IR) index, in women with PCOS, those with an isolated PCOS criterion, and a healthy population, using the FDA approach stratified by BMI and waist circumference (WC) status.

## Materials and methods

The participants of the present study were recruited from the Tehran Lipid and Glucose Study (TLGS). This study is an ongoing prospective, long-term, community-based cohort study started in 1998, mainly aimed to evaluate the factors associated with non-communicable diseases (NCDs) in a representative sample of Tehran, Iran. A total of 15,005 individuals, male and female, aged 3 years and older were included in the first phase and were followed up every 3 years. Data on various risk factors for NCDs, demographic variables, and reproductive backgrounds were collected through face-to-face interviews at the study’s onset and during follow-up visits. At each visit, comprehensive physical examinations and anthropometric assessments were performed. Furthermore, the overall anthropometric measurements and physical examinations encompassed an evaluation of hirsutism using the modified Ferriman-Gallwey scoring system, which was conducted by a general practitioner. In addition, female participants underwent an ultrasound assessment using either a 3.5 MHz transabdominal transducer for virgin individuals or a 5 MHz transvaginal transducer for others. A blood sample was collected between 07:00 and 09:00 h after 12 h of overnight fasting, during the early follicular phase of either the spontaneous or progesterone-induced menstrual cycle. Detailed descriptions of the TLGS methodology have been published elsewhere (2, 23).

The current study was approved by the ethical review board of the Research Institute for Endocrine Sciences (IR.SBMU.ENDOCRINE.REC.1404.042), and written informed consent was obtained from all participants.

### Study population

The present study included all reproductive-aged female TLGS participants (18–49 years) who completed related examinations in the first phase of the TLGS (1998–2001) and completed the three scheduled follow-up visits, with an average interval of approximately 3 years. All individuals who were pregnant, postmenopausal, or had a history of hysterectomy or bilateral oophorectomy, as well as those with endocrine disorders such as hyperprolactinemia, thyroid disorders, congenital adrenal hyperplasia, androgen-secreting neoplasms, or Cushing’s syndrome, were excluded from the current study. Furthermore, serum fasting insulin levels at the commencement of the study and at each of the three subsequent follow-up visits must be available for all participants included in the study. Finally, the remaining participants were classified into three groups: women with PCOS (*n* = 287), women with isolated PCOS criterion – only OA, only hyperandrogenemia, or only PCOM (*n* = 536), and healthy eumenorrheic, non-hirsute women without PCOM (*n* = 936). Furthermore, each group was sub-categorized based on BMI (<25 kg/m^2^, 25–29.99 kg/m^2^, and ≥30 kg/m^2^) and WC (<88 cm and ≥88 cm) measurements.

### Definition of terms

PCOS was defined according to the Rotterdam criteria (3), which require the presence of two or more of the following features: OA, clinical or biochemical hyperandrogenemia, and PCOM. OA was defined as either regular or irregular menstrual cycles lasting 34 days or longer, or a history of eight or fewer menstrual cycles per year. The clinical manifestations of hyperandrogenism included hirsutism, acne, or androgenic alopecia. Hirsutism was diagnosed using the modified Ferriman-Gallwey scale with a score of 8 or higher (24). It should be noted that since PCOS is a chronic condition with clinical manifestations that evolve across the lifespan, we performed a retrospective reclassification of the cohort using the more inclusive and contemporary 2003 Rotterdam criteria.

Acne was evaluated based on its type, quantity, and distribution (25). Biochemical hyperandrogenism was identified by elevated levels of one or more serum androgens, including dehydroepiandrosterone sulfate (DHEAS), total testosterone (TT), or androstenedione (A4), exceeding the 95th percentile compared to healthy, non-hirsute, eumenorrheic women within the study population (26). The established upper normal limits for these measures were 0.89 ng/mL for TT, 2.9 ng/mL for androstenedione, and 179 mg/dL for DHEAS.

PCOM was diagnosed by the presence of 12 or more follicles in each ovary measuring 2–9 mm in diameter and/or increased ovarian volume of more than 10 cm^3^.

BMI was determined by dividing weight in kilograms by the square of height in meters (kg/m^2^). WC was measured with an unstretched tape at the midpoint between the lower edge of the rib cage and the upper border of the iliac crest.

HOMA-IR index was calculated using the following formula: HOMA-IR = [fasting blood glucose (FBG)] (mmol/L) × [insulin] (μU/mL)/22.5.

### Biochemical and hormonal measurement

Levels of 17-hydroxyprogesterone (17OH-P), TT, A4, and DHEAS were determined by enzyme immunoassay (EIA) using kits from Diagnostic Biochem Canada Co. (Canada). Sex hormone-binding globulin (SHBG) was measured via immunoenzymometric assay (IEMA; Mercodia, Sweden). Insulin levels were assessed using electrochemiluminescence immunoassay (ECLIA) with Roche Diagnostics kits (GmbH, Germany). All measurements by radioimmunoassays, enzyme-linked immunoassays, and ECLIA methods were performed using a Dream Gamma-10 gamma counter (Shin Jin Medics, Inc., Korea), a Sunrise reader (Tecan Co., Austria), and a Roche/Hitachi Cobas e-411 analyzer (GmbH, Germany), respectively. The intra- and inter-assay coefficients of variation (CVs) were as follows: LH, 3 and 5.8%; FSH, 3.5 and 4%; 17OH-P, 4.8 and 6.8%; TT, 5.6 and 6.6%; A4, 2.2 and 3.5%; DHEAS, 2.0 and 5.1%; SHBG, 1.2 and 5.7%; and insulin, 1.2 and 3.5%, respectively.

FBG was measured using the glucose oxidase enzymatic colorimetric method with a Pars Azmoon kit (Iran), achieving intra- and inter-assay CVs of less than 2.2%. Total cholesterol (TC) was quantified using an enzymatic colorimetric method involving cholesterol esterase and cholesterol oxidase. High-density lipoprotein cholesterol (HDL-C) was assessed by precipitating apolipoprotein B-containing lipoproteins with phosphotungstic acid. Triglycerides (TG) were measured using glycerol phosphate oxidase. The intra- and inter-assay CVs for TC, HDL-C, and TG were below 1.9, 3, and 2.1%, respectively. All analyses were performed with the appropriate kits from Pars Azmoon Inc. (Iran) and a Selecta 2 autoanalyzer (Vital Scientific, Netherlands). Low-density lipoprotein cholesterol (LDL-C) was calculated using the modified Friedewald equation (27).

To monitor the accuracy of hormonal and biochemical measurements, lyophilized quality control materials (Lyphochek Immunoassay Plus Control (Bio-Rad Laboratories, USA) and TruLab N and TruLab P (Pars Azmoon Inc., Iran), respectively) in different concentration ranges were used.

### Statistical analysis

Continuous variables were tested for normality using the one-sample Kolmogorov–Smirnov test. Data were shown as the mean (standard deviation) for normally distributed variables, or as the median with interquartile range (IQR: 25th–75th percentiles) for variables with skewed distributions. Categorical variables were expressed as frequencies and percentages.

The multivariate outlier detection method was used to identify potential outliers, employing the Generalized S-Estimate (GSE) function from the GSE package. This function implements the GSE method, which can handle missing values and high-dimensional settings (https://cran.r-project.org/package=GSE) (28). The outliers for HOMA-IR and fasting insulin were detected with kernel density estimator and nearest neighbor algorithms and are shown in Supplementary Figs 1 and 2 (see section on Supplementary materials given at the end of the article), respectively. Women’s characteristics were compared between groups using the independent two-sample *t*-test (or ANOVA) for continuous variables with normal distribution, as determined by the Shapiro–Wilk test, and homogeneity of variance, as assessed by Levene’s test. If these assumptions were violated, the Kruskal–Wallis rank-sum test was applied. For post-hoc analysis, two tests were used: i) Tukey’s honest significant differences, and ii) Games-Howell post-hoc test, the latter applied when the homogeneity of variance assumption was violated. All calculations for this part were done with the rstatix package (https://cran.r-project.org/package=rstatix).

The observation curves for HOMA-IR were treated as sparse curves or sparse functional data, with each participant contributing between one and four measurements. FPCA was performed separately for each patient group, retaining the first principal components that accounted for 85% fraction of variance explained in the data with the FPCA function from the fdapace library (29). Statistical analyses were conducted using R software (version 4.4.1), with a significance level set at *P* < 0.05.

## Results

The baseline characteristics of the study participants are shown in Table 1. Women with PCOS were significantly younger than those with isolated PCOS criteria and healthy controls (mean age: 23.5 vs 25.0 and 28.0 years, respectively; all *P* < 0.05). They also had a significantly lower BMI compared to both comparison groups (23.5 vs 24.1 and 25.1 kg/m^2^, respectively; *P* < 0.05). No statistically significant differences were observed between these three groups in terms of HOMA-IR and FBG. Details of all post-hoc comparisons are shown in Supplementary Table 1. The mean (SD) BMI, WC, and weight of the healthy group across all four visits are shown in Table 2.

**Table 1:** Baseline characteristics of the study population stratified by their PCOS status.

	Healthy controls	Women with isolated PCOS criterion	Women with PCOS
	(*n* = 936)	(*n* = 536)	(*n* = 287)
Age at baseline (years), median (IQR)	28 (20)	25 (18)	23.5 (19)^†^
BMI (kg/m^2^)	25.16 (5.75)	24.11 (6.00)	23.50 (6.30)^†^
SBP (mmHg)	108 (16)	106 (15)^§^	105 (16)^‡^
DBP (mmHg)	73 (13.5)	72 (13)^§^	71 (14)^‡^
FPG (mg/dL)	87 (11)	86 (11)	86 (11)
TC (mg/dL)	183 (51)	179 (50)	177 (48.25)^‡^
LDL-C (mg/dL)	114.8 (43)	110.2 (41.8)^§^	111.3 (42.3)^‡^
HDL-C (mg/dl)	43.5 (14)	42 (13)	42 (14)
TG (mg/dL)	102 (74)	103 (72)	102 (71.25)
TyG index	9.09 (0.75)	9.08 (0.72)	9.09 (0.69)
HOMA-IR	1.733 (1.179)	1.737 (1.163)	1.765 (1.283)

Data presented as mean (standard deviation) unless otherwise indicated.

IQR, interquartile range; SD, standard deviation.

PCOS: polycystic ovary syndrome; BMI, body mass index; HOMA-IR, homeostatic model assessment for insulin resistance; TC, total cholesterol; LDL-C, low-density lipoprotein cholesterol; HDL-C, high-density lipoprotein cholesterol; DBP, diastolic blood pressure; SBP, systolic blood pressure; FPG, fasting plasma glucose; QUICKI, quantitative insulin sensitivity check index; TG, triglyceride; TyG Index, triglyceride glucose index.

^†^ Significant differences between all groups.

^‡^ Significant differences between healthy controls and women with PCOS.

^§^ Significant differences between healthy controls and women with isolated PCOS phenotype.

**Table 2:** Mean (SD) BMI, WC, and weight across four visits in healthy women, women with isolated PCOS criteria, and women with PCOS.

Group	Variable*	Visit 1	Visit 2	Visit 3	Visit 4
Healthy	BMI (kg/m^2^)	25.16 (5.75)	26.52 (5.56)	27.09 (5.29)	28.07 (5.21)
	WC (cm)	80.67 (14.54)	84.61 (14.11)	84.76 (13.61)	90.51 (12.42)
	Weight (kg)	61.06 (17.13)	64.67 (14.91)	66.59 (13.44)	68.41 (12.55)
Isolated PCOS criteria	BMI	24.11 (6.00)	25.83 (5.72)	26.44 (5.44)	27.41 (5.63)
	WC (cm)	77.83 (14.81)	82.25 (13.92)	82.47 (13.89)	88.60 (13.02)
	Weight (kg)	58.07 (18.53)	63.17 (15.91)	65.55 (14.07)	67.47 (13.60)
PCOS	BMI	23.50 (6.30)	25.40 (6.04)	26.25 (5.73)	27.33 (5.78)
	WC (cm)	76.06 (15.08)	81.00 (14.70)	82.09 (13.63)	88.39 (13.02)
	Weight (kg)	56.17 (20.30)	62.13 (17.29)	65.39 (14.86)	68.03 (13.91)

* Mean (SD).

BMI, body mass index; WC, waist circumference.

### HOMA-IR trajectories by BMI

**Figure 1:**
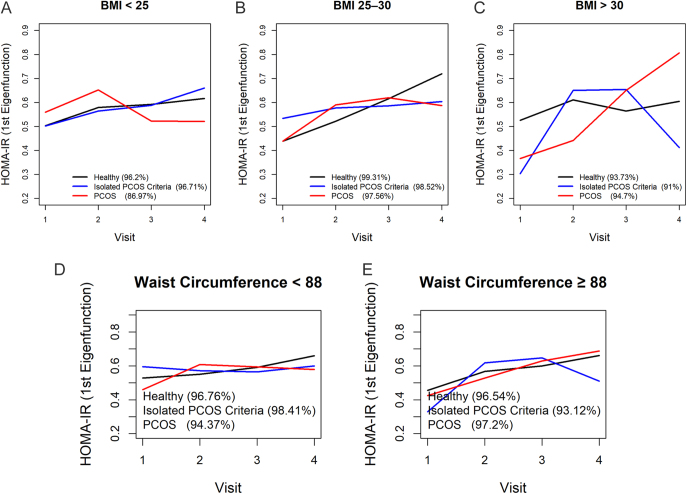
The first eigenfunctions of HOMA-IR by BMI (A) BMI <25 kg/m^2^, (B) BMI: 25–30 kg/m^2^, (C) BMI > 30 kg/m^2^), (D) WC < 88 cm, and (E) WC ≥ 88 cm in different groups. BMI: body mass index; WC: waist circumference; HOMA-IR: homeostatic model assessment for insulin resistance.

Figure 1A, B, C presents the first eigenfunctions of HOMA-IR stratified by BMI subgroups: i) normal weight, ii) overweight, and iii) obese.

- In normal weight women, all three groups maintained relatively stable HOMA-IR values across visits, with only modest upward trends. The PCOS group started slightly above the others at visit 1, peaked between visits 2 and 3, and plateaued by visit 4, suggesting an initial worsening of IR followed by stabilization. The isolated criterion and healthy groups showed overlapping, subtle increases without sharp divergence, indicating minimal BMI-related IR progression in this lean cohort.
- In the overweight subgroup, differences emerged. The healthy group exhibited a linear upward trend across visits, while the isolated criterion group closely followed a similar pattern with a slight lag. The PCOS group started lower at the beginning but converged by visit 2, paralleling the healthy and isolated criterion trajectories between visits 3 and 4, indicating that overweight women with PCOS experienced IR increases comparable to their peers in this BMI range.
- Women with obesity displayed the most divergent trajectories. The PCOS group showed a continuous, steep rise in HOMA-IR from visit 1 to visit 4, reflecting progressively worsening IR. In contrast, the isolated criterion group started lower and declined steadily through visit 4, suggesting an improvement in insulin sensitivity over time. The healthy group exhibited a moderate increase until visit 3, followed by a slight dip at visit 4, positioning it between the other groups.

**Figure 2:**
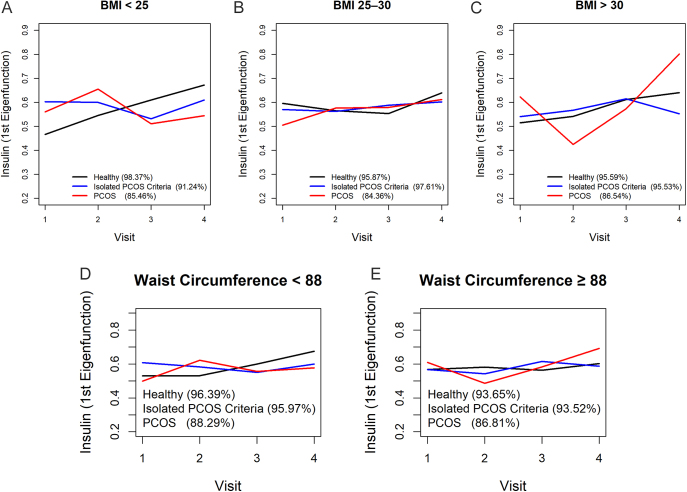
The first eigenfunctions of fasting insulin concentration by BMI (A) BMI <25 kg/m^2^, (B) BMI: 25–30 kg/m^2^, (C) BMI > 30 kg/m^2^, (D) WC < 88 cm, and (E) WC ≥ 88 cm in different groups. BMI: body mass index; WC: waist circumference; HOMA-IR: homeostatic model assessment for insulin resistance.

### HOMA-IR trajectories by WC

Figure 1D and E present the first eigenfunctions of HOMA-IR for two WC groups: iv) WC < 88 cm and v) WC ≥ 88 cm.

- In women with WC < 88 cm, all groups (PCOS, isolated criterion, and healthy) maintained relatively stable HOMA-IR values across visits, with only modest upward trends. The PCOS group started slightly above the others at visit 1, peaked between visits 2 and 3, and plateaued by visit 4, suggesting an initial worsening of IR followed by stabilization. The isolated criterion and healthy groups showed overlapping, subtle increases without sharp divergence, indicating minimal WC-related IR progression in this cohort with lower central adiposity.
- In women with WC ≥ 88 cm, differences became more pronounced. The healthy group exhibited a linear upward trend across visits, reflecting a gradual increase in IR with higher WC. The isolated criterion group closely followed a similar pattern with a slight lag, suggesting a comparable metabolic response. The PCOS group started lower at visit 1 but converged by visit 2, paralleling the healthy and isolated criterion trajectories between visits 3 and 4, indicating that women with PCOS and increased WC experienced IR increases similar to their peers in this higher WC range. The steeper trajectory in this group highlighted the exacerbating effect of central obesity on IR, particularly in PCOS.

### Fasting serum insulin trajectories by BMI

Figure 2A, B, C present the first eigenfunction trajectories of fasting serum insulin across four visits, stratified by BMI: i) normal weight, ii) overweight, and iii) obese.

- Normal weight women exhibited stable and overlapping IR trajectories with minor early fluctuations in PCOS and isolated‐criterion participants. The PCOS group started marginally higher at visit 1, peaked between visits 2 and 3, and stabilized by visit 4, suggesting an early decline in insulin sensitivity followed by recovery. The isolated‐criterion and healthy groups remained almost parallel, demonstrating only slight variability and minimal BMI-related changes in IR within this subgroup.
- In overweight participants, trajectories were more synchronized across groups. The healthy group showed a gentle upward trend in IR across visits. The isolated‐criterion group followed closely with only a minor delay. The PCOS group began lower but converged by visit 2, aligning with the other two groups through visits 3 and 4. This convergence indicates that overweight women with PCOS exhibited IR increases comparable to their healthy and isolated‐criterion counterparts.
- In obese women, trajectories diverged markedly. The PCOS group displayed a continuous, steep increase in IR from visit 1 to visit 4, signaling progressively worsening metabolic dysfunction. The isolated‐criterion group began lower but showed a consistent decrease over time, indicative of improved insulin sensitivity. The healthy group experienced a moderate increase up to visit 3, followed by a slight decline at visit 4, positioning it between the other two groups.

### Fasting serum insulin trajectories by WC

Figure 2D and E present the first‐eigenfunction trajectories of fasting serum insulin concentration across four visits, stratified by WC: i) WC < 88 cm (normal abdominal size) and ii) WC ≥ 88 cm (central obesity).

- In women with WC < 88 cm, serum insulin trajectories were stable and showed only moderate variation across groups. Healthy and isolated-criterion groups began at similar levels and displayed gentle, parallel increases through visit 4. The PCOS group started slightly lower, rose modestly through visits 2–3, and plateaued by visit 4, suggesting a minor early change before stabilizing. This finding may show that in the absence of central obesity, IR progresses modestly and similarly across PCOS and non-PCOS groups.
- In women with central obesity (WC ≥ 88 cm), where abdominal adiposity is elevated, serum insulin trajectories diverged markedly. The healthy group showed a steady increase from visit 1 to visit 4. The isolated-criterion group began lower, experienced a steeper rise peaking at visit 3, then dipped at visit 4, indicating early worsening followed by improvement. The PCOS group started at a higher level than others, climbed past both other groups by visit 3, and continued rising by visit 4, signifying ongoing worsening of IR.

## Discussion

This population-based longitudinal study applied FDA to present novel insights into the dynamic patterns of IR by identifying distinct HOMA-IR and serum insulin concentration trajectories among women with PCOS, those meeting an isolated PCOS criterion, and healthy controls, stratified based on general or central obesity. The findings revealed two key patterns: i) women with PCOS demonstrated upward IR trajectories over time. However, these trajectories varied by general and central obesity status. Among women with PCOS without obesity or central obesity, HOMA-IR trajectories initially increased but showed a mild and steady decline over time, suggesting a potential improvement in IR. In contrast, women with PCOS and either obesity or central obesity showed a sharp and continuous rise in HOMA-IR values, indicating a progressive worsening of IR. ii) Women in the healthy and isolated PCOS criterion groups without general or central obesity generally exhibited stable or only moderately increasing HOMA-IR trajectories with less pronounced changes. Among healthy individuals with general or central obesity, HOMA-IR increased moderately over time, though the progression was less steep than that observed in the PCOS group. However, women with an isolated PCOS criterion with general or central obesity showed decreasing trajectories, particularly in later follow-up, exceeding the lowest level at the end of follow-up.

IR refers to a pathological condition in which insulin-targeting tissues exhibit a reduced responsiveness to normal circulating insulin levels. This diminished sensitivity disrupts metabolic processes regulated by insulin and affects the biological actions of insulin, including glucose, lipid, and protein metabolism, as well as vascular function and gene regulation (30). Although the mechanism of IR has not been fully established, it is suggested that impaired insulin receptor signaling pathways, chronic low-grade inflammation, mitochondrial dysfunction, and lipid accumulation in non-adipose tissues, all of which may impair the normal cellular response to insulin and contribute to systemic metabolic dysregulation (31, 32).

IR and the resulting compensatory hyperinsulinemia are central to the pathophysiology of PCOS (6, 33), influencing both metabolic and reproductive functions (5). In addition, IR in PCOS is associated with an elevated risk of long-term metabolic disorders, such as type 2 diabetes, dyslipidemia, and cardiovascular disease (8, 34, 35). It is reported that it affects 65–95% of women with the syndrome, including the vast majority of overweight and obese women, as well as more than half of women of normal weight (36, 37, 38).

Weight gain or fat accumulation, particularly when associated with BMI, and the accumulation of visceral fat, as indicated by WC, are well-established contributors to the progression of IR, exacerbating insulin signaling impairments and promoting a pro-inflammatory state (39, 40). In this respect, Park *et al.* during 20 years of follow-up reported that WC and long-term increments in WC are associated with worsening IR (40). In another study, Walsh *et al.* examined the impact of body weight and preceding weight change trajectories over 12 years on blood glucose, insulin, and HOMA2 measures of insulin metabolism. Their results showed that high body weight is associated with elevated blood glucose levels and impaired insulin function. Of much greater interest, weight change is associated with insulin metabolism in adult humans, and trajectories of weight gain and high weight maintenance have distinguishable impacts on insulin metabolism. They concluded that the trajectory of preceding weight has an independent effect on blood glucose metabolism beyond body weight measured at any given point in time (41).

Our findings showed that women with PCOS but without general or central obesity exhibited an initial upward trajectory in HOMA-IR during earlier follow-ups, followed by a modest decline and improvement in later years. We hypothesize that this decline may be partially attributed to age-related changes, including the natural decline in the number of ovarian follicles and diminished capacity for ovarian steroid secretion (42, 43), as well as a significant age-related decrease in adrenal androgen production, such as DHEAS (44). In line with this finding, several longitudinal studies have reported an amelioration of PCOS phenotypic characteristics with aging, as indicated by improved cycle regularity and symptoms of hyperandrogenism (45, 46, 47). In addition, increased health awareness or clinical management in older age, including lifestyle modifications, may contribute to reduced IR.

In contrast, in individuals with general or central obesity, the PCOS group exhibits a pronounced increase in HOMA-IR over time, indicating a progressive deterioration in IR. We hypothesize that this trajectory may be driven by the synergistic effects of excess adiposity and the underlying metabolic disturbances characteristic of PCOS. Adipose tissue, particularly visceral fat, is known to contribute to chronic low-grade inflammation and the secretion of adipokines that impair insulin signaling. Moreover, the persistence of hyperandrogenemia in women with PCOS may exacerbate adipocyte dysfunction and further impair glucose homeostasis. The combination of these factors may create a self-reinforcing cycle of worsening IR (48). Supporting this hypothesis, a meta-analysis of 27 observational studies demonstrated that PCOS status exacerbates the adverse effect of obesity on IR (6).

In addition, age-related declines in physical activity and increasing fat accumulation with time may contribute to the progressive trajectory observed in this group.

In addition, we found that in women without general or central obesity, both the isolated PCOS criterion and healthy groups exhibited relatively stable, gradually increasing HOMA-IR trajectories over time. This pattern may reflect the well-established, age-related decline in insulin sensitivity that occurs even in metabolically healthy individuals due to physiological changes such as reduced muscle mass, increased visceral fat deposition, and alterations in mitochondrial function and insulin signaling pathways with aging (49). However, in the healthy group with general or central obesity, we observed a pronounced upward trend in HOMA-IR levels, indicating a progressive worsening of IR. This is likely attributable to the synergistic effect of aging and adiposity-related mechanisms, including chronic inflammation, ectopic fat accumulation, adipokine imbalance, and impaired insulin receptor signaling. These factors can accelerate the deterioration of glucose homeostasis and insulin sensitivity over time (50).

In contrast, the isolated PCOS criterion group with general or central obesity exhibited a distinct HOMA-IR trajectory, characterized by an initial increase followed by a noticeable decline in the later phases of follow-up. This pattern diverges from what is typically observed in classic PCOS and raises several possible explanations. One hypothesis is that some women in this group may carry genetic or epigenetic variants that provide partial protection against the full development of PCOS and its associated metabolic dysfunctions. For example, recent studies have identified loci associated with both susceptibility and resilience to PCOS features, including IR and hyperandrogenism (51, 52). It is possible that women with an isolated diagnostic feature, particularly when it does not progress to meet full diagnostic criteria, may represent a genetically distinct subgroup with a more favorable metabolic profile over time. Furthermore, these individuals may have a milder endocrine phenotype, with only transient or subclinical disruptions in ovarian or adrenal function, which may resolve or attenuate with age. The age-related decline in androgen production, combined with possible compensatory improvements in insulin signaling, may contribute to the observed decline in HOMA-IR during later follow-ups. In addition, increased healthcare engagement, awareness of metabolic risks, and adoption of healthier lifestyles in midlife may further reduce IR in this group.

However, it should be noted that the HOMA-IR values between individuals with PCOS and those who are healthy, or those meeting only an isolated PCOS criterion, are relatively modest in this population-based study. This is likely due to the inclusive nature of the cohort, which captures a wide spectrum of phenotypic expression, including milder or subclinical presentations. Consequently, differences between groups may be attenuated compared to clinical samples, where individuals typically present with more severe symptoms.

Similar to HOMA-IR trajectories, fasting serum insulin, a key determinant of HOMA-IR, serves as a sensitive indicator of compensatory hyperinsulinemia, which is central to the pathophysiology of IR in PCOS. Our findings reveal that, in women with WC < 88 cm, serum insulin trajectories remained largely stable and overlapping across healthy, isolated-criterion, and PCOS groups, suggesting that, in the absence of central adiposity, hyperinsulinemia in PCOS may be driven more by intrinsic defects in insulin signaling than by excess visceral fat. Conversely, in women with WC ≥ 88 cm, PCOS cases exhibited a persistent and steep rise in fasting insulin from baseline to final visit, which may show the pathological role of visceral adiposity that impairs insulin signaling.

In addition, we observed that women with PCOS in this study have a lower BMI (23.38 kg/m^2^) than healthy women (24.46 kg/m^2^, *P* < 0.05), which contrasts with most studies that typically report higher BMI in PCOS. This observation may be attributed to the community-based recruitment in this study, which likely included a higher proportion of the lean PCOS phenotype that often presents with normal BMI despite significant metabolic dysfunction (53). As such, the significantly younger age of women with PCOS compared to non-PCOS groups may also play a potential role. Furthermore, the use of the Rotterdam criteria for PCOS diagnosis might include milder cases, thereby diluting the association with obesity.

It should be noted that we considered IR trajectories by obesity status because obesity is a key modifiable risk factor that plays a central role in the development and progression of IR, particularly in women with PCOS. There is strong evidence that both the presence and degree of obesity exacerbate IR in this population. By stratifying trajectories based on obesity status, we aimed to capture potential heterogeneity in the evolution of IR over time. In addition, we selected BMI and WC as indicators of obesity because they are the most widely used and validated anthropometric measures for assessing general and central adiposity, respectively. BMI is a standard measure for overall obesity, while WC provides a more specific estimate of abdominal fat distribution, which is strongly associated with metabolic dysfunction and IR. By including both, we were able to assess whether WC, which has a closer physiological link to IR, provides additional insight beyond overall adiposity measured by BMI in shaping IR trajectories. This dual approach allowed us to examine how different types of adiposity influence the longitudinal course of IR in women with PCOS and potentially identify high-risk phenotypes for earlier intervention.

The identification of distinct IR trajectories in women with PCOS, particularly those with general and central obesity, has important clinical implications for risk stratification and personalized care. Given the progressive worsening of IR observed in women with obesity and PCOS, targeted lifestyle modifications, nutritional counseling, and, when appropriate, pharmacological strategies may be prioritized early in the clinical course to prevent long-term cardiometabolic complications. The observed improvement in IR among non-obese PCOS women over time also suggests that weight-independent factors, including aging, endocrine normalization, or increased health engagement, may positively influence metabolic trajectories, supporting the value of longitudinal, individualized management plans in PCOS care.

Our study has several notable strengths. To the best of our knowledge, this is the first study to present distinct trajectories of IR in the context of PCOS. In addition, the application of FDA with FPCA allowed us to model sparse longitudinal data as continuous trajectories, uncovering nuanced patterns that traditional statistical methods might overlook. The stratification of participants by BMI and WC also provided valuable insights into the heterogeneity of IR trajectories across different general and central obesity statuses. Furthermore, the comprehensive data collection within the TLGS, including anthropometric assessments, ultrasound evaluations, and blood sampling during the early follicular phase, ensured standardized and reliable measurements of PCOS criteria. Moreover, insulin was measured by ECLIA, a sensitive method with good analytical specificity (54). Finally, the large sample size, extended follow-up period, and the inclusion of both PCOS and isolated PCOS criterion groups alongside healthy controls enabled us to provide novel insights into the spectrum of these conditions. A number of limitations should be considered when interpreting the results of this study. FPCA assumes smoothness in the data; however, our dataset is characterized by sparse and irregularly observed measurements. To decrease this issue, we applied FPCA using the fdapace package in R, which is specifically designed for the analysis of sparse functional data. The lack of detailed data on specific interventions, such as pharmacological treatments or lifestyle modifications in different groups, limits our ability to determine their direct impact on the observed HOMA-IR trajectories. In addition, while FPCA effectively captured the variance in HOMA-IR trajectories, the remaining unexplained variance suggests that other unmeasured factors, such as genetic or epigenetic variants, may also shape IR dynamics. Furthermore, we used ultrasound assessments with a 3.5 MHz transabdominal transducer for virgin individuals or a 5 MHz transvaginal transducer for others, which may compromise the resolution and accuracy of PCOM detection, potentially leading to underdiagnosis or misclassification of PCOS. Moreover, FPCA assumes smoothness in the data; however, our dataset is characterized by sparse and irregularly observed measurements. To decrease this issue, we applied FPCA using the fdapace package in R, which is specifically designed for the analysis of sparse functional data. Finally, the study population was drawn from the TLGS, which is specific to a Persian cohort, potentially limiting the generalizability of the findings to other ethnic groups with different genetic and environmental risk profiles for IR and PCOS.

In conclusion, our study provides novel insights into the distinct trajectories of IR in women with PCOS, those meeting an isolated PCOS criterion, and healthy controls. Women with PCOS, particularly those with general or central obesity, showed a progressive worsening of IR, while those without obesity exhibited a potential improvement over time. In contrast, the healthy and isolated PCOS criterion groups exhibited more stable or moderate increases in IR, with a less pronounced progression. These findings emphasize the importance of considering general and central obesity status in understanding IR dynamics in women with PCOS and suggest that early interventions targeting these factors may help mitigate the risk of long-term metabolic complications.

## Supplementary materials



## Declaration of interest

The authors declare that this study was conducted in the absence of any commercial or financial relationships that could be construed as potential conflicts of interest.

## Funding

This study was funded by the Research Institute for Endocrine Sciences, Shahid Beheshti University of Medical Scienceshttps://doi.org/10.13039/501100005851, Tehran, Iran (Grant number: 5-43015947). Nord University covered the article processing charge.

## Author contribution statement

FRT and SB-G contributed to the conceptualization, formal analysis, and methodology of the study, wrote the original draft, and participated in the review and editing process. MF performed formal analysis and investigation and contributed to writing the original draft. FF and FA participated in investigation, review, and editing. MT participated in investigation, laboratory analyses, review, and editing. All authors approved the final version of the study.
