# Supplementary material for: Longitudinal trajectories of insulin resistance in women with polycystic ovary syndrome: a functional data analysis approach
Source: Endocr Connect. 2025 Aug 29;14(8):e250266. doi: 10.1530/EC-25-0266 (PMC12400798; doi:10.1530/EC-25-0266)
Supplement: Supplementary file 1 [file supplementary_materials.pdf]

Supplementary material

Supplementary Table 1 – The Post-Hoc Comparison with Tukey-HSD methods

Variable	Group1	Group2	Mean Difference Estimate	Confidence Interval Lower Bound	Confidence Interval Upper Bound	Adjusted P-value	Adjusted P-value Significance
Age	Healthy Controls	Isolated PCOS criterion	-3.087	-4.066	-2.107	0.000	****
	Healthy Controls	PCOS	-5.001	-6.221	-3.782	0.000	****
	Isolated PCOS criterion	PCOS	-1.915	-3.231	-0.598	0.002	**
Body Mass Index	Healthy Controls	Isolated PCOS criterion	-0.684	-1.158	-0.210	0.002	**
	Healthy Controls	PCOS	-1.125	-1.715	-0.535	0.000	****
	Isolated PCOS criterion	PCOS	-0.441	-1.077	0.195	0.023	**
Total Cholesterol	Healthy Controls	Isolated PCOS criterion	-3.252	-6.550	0.045	0.054	non-significant
	Healthy Controls	PCOS	-4.749	-8.851	-0.647	0.018	*
	Isolated PCOS criterion	PCOS	-1.497	-5.922	2.928	0.707	non-significant
Diastolic blood pressure	Healthy Controls	Isolated PCOS criterion	-1.127	-1.954	-0.301	0.004	**
	Healthy Controls	PCOS	-1.571	-2.601	-0.542	0.001	**
	Isolated PCOS criterion	PCOS	-0.444	-1.555	0.667	0.617	non-significant
Fasting Plasma Glucose	Healthy Controls	Isolated PCOS criterion	-0.725	-1.590	0.140	0.121	non-significant
	Healthy Controls	PCOS	-0.751	-1.827	0.325	0.230	non-significant
	Isolated PCOS criterion	PCOS	-0.026	-1.186	1.134	0.998	non-significant
HDL-C: High-Density Lipoprotein Cholesterol	Healthy Controls	Isolated PCOS criterion	-0.225	-1.142	0.693	0.834	non-significant
	Healthy Controls	PCOS	-0.760	-1.901	0.382	0.263	non-significant
	Isolated PCOS criterion	PCOS	-0.535	-1.767	0.697	0.565	non-significant
HOMA_IR_index	Healthy Controls	Isolated PCOS criterion	-0.007	-0.141	0.126	0.991	non-significant
	Healthy Controls	PCOS	0.094	-0.072	0.260	0.382	non-significant
	Isolated PCOS criterion	PCOS	0.101	-0.079	0.281	0.386	non-significant
LDL-C: Low-Density Lipoprotein Cholesterol	Healthy Controls	Isolated PCOS criterion	-2.952	-5.776	-0.128	0.038	*
	Healthy Controls	PCOS	-3.892	-7.408	-0.376	0.026	*
	Isolated PCOS criterion	PCOS	-0.940	-4.731	2.851	0.830	non-significant
Systolic blood pressure Triglyceride Glucose Index	Healthy Controls	Isolated PCOS criterion	-1.734	-2.828	-0.640	0.001	***
	Healthy Controls	PCOS	-2.646	-4.009	-1.283	0.000	****
	Isolated PCOS criterion	PCOS	-0.912	-2.382	0.559	0.314	non-significant
	Healthy Controls	Isolated PCOS criterion	-0.021	-0.068	0.026	0.542	non-significant
	Healthy Controls	PCOS	-0.020	-0.078	0.038	0.686	non-significant

	Isolated PCOS criterion	PCOS	0.001	-0.062	0.063	1.000	non-significant

Table S2 – The Post-Hoc Comparison with Games Howell methods

Variable	Group1	Group 2	Mean Difference Estimate	Confidence Interval Lower Bound	Confidence Interval Upper Bound	Adjusted P-value	Adjusted P-value Significance
Age	Healthy Controls	Isolated PCOS criterion	-3.087	-4.064	-2.109	0.000	****
	Healthy Controls	PCOS	-5.001	-6.182	-3.820	0.000	****
	Isolated PCOS criterion	PCOS	-1.915	-3.177	-0.652	0.001	***
Body Mass Index	Healthy Controls	Isolated PCOS criterion	-0.684	-1.156	-0.213	0.002	**
	Healthy Controls	PCOS	-1.125	-1.741	-0.509	0.000	****
	Isolated PCOS criterion	PCOS	-0.441	-1.106	0.224	0.0266	**
Total Cholesterol	Healthy Controls	Isolated PCOS criterion	-3.252	-6.511	0.006	0.051	non-significant
	Healthy Controls	PCOS	-4.749	-8.909	-0.589	0.020	*
	Isolated PCOS criterion	PCOS	-1.497	-5.927	2.934	0.708	non-significant
Diastolic Blood Pressure	Healthy Controls	Isolated PCOS criterion	-1.127	-1.950	-0.305	0.004	**
	Healthy Controls	PCOS	-1.571	-2.603	-0.539	0.001	***
	Isolated PCOS criterion	PCOS	-0.444	-1.550	0.662	0.614	non-significant
Fasting Plasma Glucose	Healthy Controls	Isolated PCOS criterion	-0.725	-1.595	0.145	0.124	non-significant
	Healthy Controls	PCOS	-0.751	-1.714	0.213	0.161	non-significant
	Isolated PCOS criterion	PCOS	-0.026	-1.052	1.000	0.998	non-significant
HDL-C: High-Density Lipoprotein Cholesterol	Healthy Controls	Isolated PCOS criterion	-0.225	-1.153	0.704	0.838	non-significant
	Healthy Controls	PCOS	-0.760	-1.888	0.369	0.255	non-significant
	Isolated PCOS criterion	PCOS	-0.535	-1.768	0.698	0.566	non-significant
HOMA_IR_index	Healthy Controls	Isolated PCOS criterion	-0.007	-0.138	0.123	0.990	non-significant
	Healthy Controls	PCOS	0.094	-0.083	0.270	0.426	non-significant

	Isolated PCOS criterion	PCOS	0.101	-0.088	0.290	0.419	non-significant
LDL-C: Low-Density Lipoprotein Cholesterol	Healthy Controls	Isolated PCOS criterion	-2.952	-5.759	-0.145	0.037	*
	Healthy Controls	PCOS	-3.892	-7.412	-0.373	0.026	*
	Isolated PCOS criterion	PCOS	-0.940	-4.704	2.824	0.828	non-significant
Systolic blood pressure	Healthy Controls	Isolated PCOS criterion	-1.734	-2.821	-0.647	0.001	***
	Healthy Controls	PCOS	-2.646	-4.003	-1.289	0.000	****
	Isolated PCOS criterion	PCOS	-0.912	-2.362	0.539	0.304	non-significant
Triglyceride Gucose Index	Healthy Controls	Isolated PCOS criterion	-0.021	-0.068	0.026	0.544	non-significant
	Healthy Controls	PCOS	-0.020	-0.078	0.037	0.679	non-significant
	Isolated PCOS criterion	PCOS	0.001	-0.061	0.062	1.000	non-significant

Supplementary Figure1: Outliers based on KDE (left) and NN (right) in step 1 for **HOMA-IR Index**

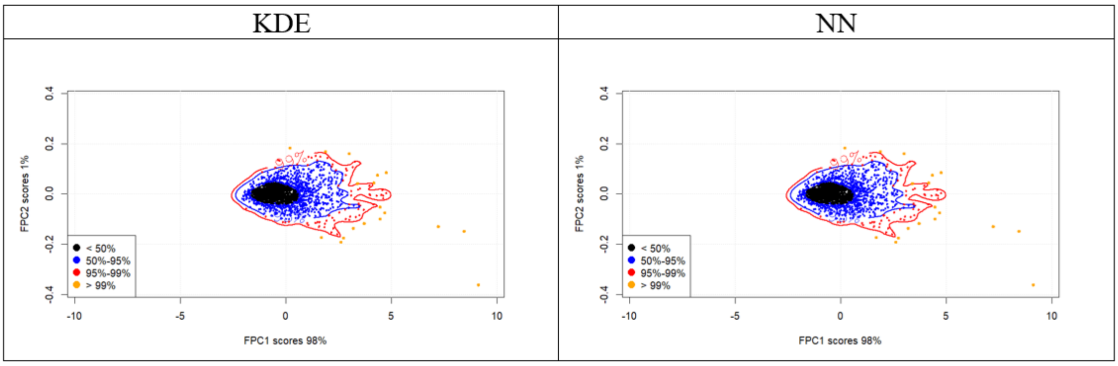

Supplementary Figure2: Outliers based on KDE (left) and NN (right) in step 1 for **Fasting Insulin**

<table>
<tr><th>KDE</th><th>NN</th></tr>
<tr><td>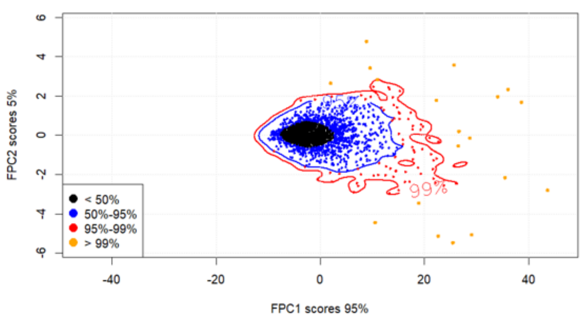
</td><td>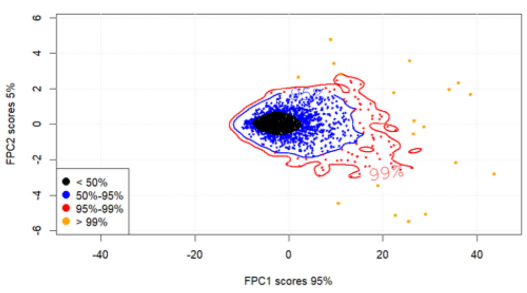
</td></tr>
</table>
